# Recent progress in studies of factors that elicit pancreatic β-cell expansion

**DOI:** 10.1007/s13238-014-0123-3

**Published:** 2014-12-11

**Authors:** Qiu Li, Zhi-Chun Lai

**Affiliations:** 1 Shandong Provincial Hospital Affiliated to Shandong University, Jinan, 250021 China; 2 Department of Biology, The Pennsylvania State University, University Park, PA 16802 USA; 3 Department of Biochemistry and Molecular Biology, The Pennsylvania State University, University Park, PA 16802 USA

**Keywords:** pancreatic islet, β-cell, cell proliferation/replication/expansion, cell signaling

## Abstract

The loss of or decreased functional pancreatic β-cell is a major cause of type 1 and type 2 diabetes. Previous studies have shown that adult β-cells can maintain their ability for a low level of turnover through replication and neogenesis. Thus, a strategy to prevent and treat diabetes would be to enhance the ability of β-cells to increase the mass of functional β-cells. Consequently, much effort has been devoted to identify factors that can effectively induce β-cell expansion. This review focuses on recent reports on small molecules and protein factors that have been shown to promote β-cell expansion.

## INTRODUCTION

The prevalence of the diabetic population in the United States is 29.1 million or 9.3% of the total population as shown in a recent National Diabetes Statistics Report (CDC, 2014). In China, the overall prevalence of diabetes is 11.6% in the adult population (Xu et al., 2013). Clearly diabetes is becoming a serious worldwide health problem as currently 350 million individuals in the world suffer from diabetes (Vetere et al., 2014). The lack of functional pancreatic β-cells leads to diabetes. Therefore, a better understanding of how an appropriate number of functional β-cells is generated and maintained shall help develop strategies for diabetic treatment. Three major approaches for increasing β-cell mass involve induction of β-cell proliferation, enhancement of β-cell viability and β-cell reprogramming (Vetere et al., 2014). This review intends to focus on regulation of β-cell proliferation. While some studies indicate that adult β-cell replication or neogeneration was hard to detect (Gunasekaran et al., 2012; Guardado-Mendoza et al., 2012; Cavelti-Weder et al., 2013; Xiao et al., 2013a, b), an increase of β-cell mass has been reported under a non-diabetic obesity condition (Klöppel et al., 1985), and in other studies in animals (Hull et al., 2005; Bock et al., 2003) and in humans (Heit et al., 2006; Rahier et al., 2008; Hanley et al., 2010; Saisho et al., 2013). During pregnancy, an increase of β-cell mass was also observed, which produced more insulin to set off insulin resistance (Sorenson and Brelje, 1997; Toselli et al., 2014). Moreover, adult β-cell proliferation was found in pancreas impaired through pancreatectomy and partial duct ligation (Dor et al., 2004; Peshavaria et al., 2006; Nir et al., 2007; Xiao et al. 2013a, b). Therefore, adult β-cells appear to be still capable of proliferation. Finding ways to enhance such capacity to expand β-cell mass shall provide important strategies for diabetic treatment.

**Figure 1:**
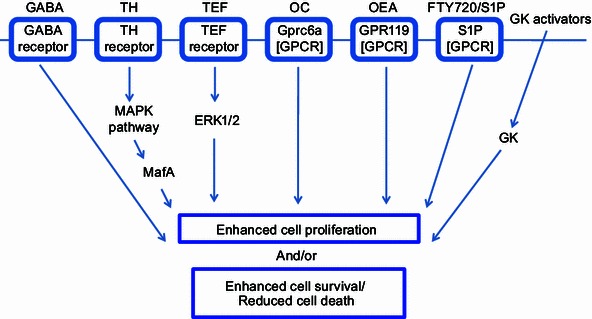
**Several factors summarized in this review act through intracellular pathways to elicit β-cell expansion by enhancing cell proliferation and/or reducing cell death**. In the case of FTY720, its effect on β-cells appears to be mediated through activation of immune cells in the lymph nodes. For the most part, molecular mechanisms of these pathways remain to be fully elucidated. GABA: γ-aminobutyric acid; OC: osteocalcin; OEA: oleoylethanolamide; S1P: sphingosine-1-phosphate; TEF: trefoil factor; TH: thyroid hormone; GPCR: G protein-coupled receptor. Gprc6a: the G protein-coupled receptor family C group 6 protein; GK: glucokinase. GK activators are small chemical compounds

Previous studies have led to the identification of many chemical compounds and biological factors that can increase β-cell mass and some recent reviews have looked into β-cell proliferation control through intracellular signaling (e.g. Kulkarni et al., 2012; Bernal-Mizrachi et al., 2014). This review focuses on small molecules and protein factors that are known to have an effect on the induction of β-cell expansion but their mechanisms of action still require further investigations (Fig. 1).

### γ-AMINOBUTYRIC ACID

The γ-aminobutyric acid (GABA) is a product derived from the amino acid glutamate (Fenalti et al., 2007). Extracellular glutamate enters β-cells through the glutamate transporter-1 (GLT-1), and is then converted to GABA (Adeghate and Ponery, 2002). GABA can be secreted by β-cells and acts as an autocrine through a positive feedback loop in pancreatic β-cells (Braun et al., 2010). It is reported that inflammation can increase β-cell proliferation (Sherry et al., 2006), and GABA could inhibit inflammation which probably partially counteracted its ability to induce proliferation (Song and Park, 2014). However, Tian et al. (2013) observed that GABA increased β-cell mass through enhanced survival and proliferation of β-cells. In rodents, GABA increased β-cell proliferation and suppressed apoptosis through up-regulated BCL-XL and dampened Caspase-3 (Ligon et al., 2007). Excitingly, it has been reported that GABA restores β-cell mass and reverse diabetes in GABA-injected mice (Soltani et al., 2011).

### THYROID HORMONE

The relationship between iodothyronine and diabetes has been studied since 1980’s. Decreased serum T3 was observed after insulin was withdrawn in juvenile type diabetic patients (Madsbad et al., 1981). It was reported that thyroid hormones reduce glucose tolerance both in animals and in humans (Lenzen and Bailey, 1984). To determine whether thyroid hormones play a role in β-cell proliferation, Ximenes et al. (2007) reported that high concentration of T3 (>250 µmol/L) attenuated β-cell proliferation, increased apoptosis and decreased the secretion of insulin, while physiological concentrations of T3 have no negative effects on the function and survival of β-cells. However, more studies have shown that thyroid hormone plays a positive role in regulating β-cells mass. T3 has been shown to reduce the risk of type 1 diabetes in autoimmune prone BB rats (Hartoft-Nielsen et al., 2009). Furthermore, T3 treatment increased β-cell mass in Wistar rats. T3 has also been shown to induce the proliferation of β-cell through the MAPK pathway and enhance the secretion of insulin (Kim et al., 2014). Aguayo-Mazzucato et al. (2013) reported that thyroid hormone promotes β-cell development through Mafa, which is a key transcription factor of β-cell differentiation (Kaneto et al., 2008; 2009). Rat pancreatic β-cell lines (RIN5F) treated with T3 induced cell proliferation, and further research shows the Cyclin D1/CDK/Rb/E2F pathway was involved in the process of β-cell replication. Furthermore, intra pancreatic injection of TRα led to an expansion of the β-cell mass in diabetic mice (Furuya et al., 2013). Moreover, ligand-bound thyroid hormone receptor TRα is involved in the reprogramming of pancreatic acinar cells into insulin-producing cells via activation of PI3K signaling (Furuya et al., 2013).

### TREFOIL FACTORS

Trefoil factors 2/3 (TFF2 and TFF3) are members of the trefoil family which are expressed in gastrointestinal mucosa. TFF3 is expressed in fetal human and newborn rat pancreas. Strong expression of TFF3 was found in islets and some pancreatic duct cells. There was significant expression of TFF3 mRNA in human islet samples examined. Exogenous TFF3 promoted islet cell attachment and migration, but had no effect on proliferation (Jackerott et al., 2006). However, over-expression of TFF3 in a RIN cell line and rat islets using recombinant adenovirus lead to an increased proliferation which can be arrested by knockdown of TFF3 using siRNA. Mediated by SDF-1α/CXCR4 signaling, another family member TFF2 promotes cell proliferation through increasing ERK1/2 phosphorylation in rat INS-1 cells, mouse MIN6 cells, and mouse islets (Orime et al., 2013). As TFF2 is not expressed in pancreatic islets (Jackerott et al., 2006), circulating TFF2 may enhance pancreatic β-cell expansion by interacting with its receptor. The serum TFF2 levels are reportedly increased during pregnancy (Samson et al., 2008; 2011). Thus, TFF2 might contribute to pancreatic β-cell expansion during pregnancy.

### OSTEOCALCIN

Osteocalcin (OC) is a Vitamin K-dependent protein secreted in the late differentiation stage of osteoblasts. It has been considered as an endocrine hormone because of its ability to reduce insulin sensitivity, decrease fat mass, and induce release of glucagon-like peptide-1 and thereby stimulate insulin secretion (Lee et al., 2007; Ferron et al., 2008; Mizokami et al., 2013). Through a molecular genetic approach, Wei et al (2014) have recently found that osteocalcin stimulates β-cell proliferation in the pancreas via a Cyclin D1-dependent mechanism utilizing the G protein-coupled receptor family C group 6 protein (Gprc6a). This stimulation occurs during the peak of β-cell proliferation, which occurs in the perinatal period, and in adult mice. Moreover, they described the effects of daily osteocalcin injections in obese type 2 diabetic mice, reporting an increase in the number of mitochondria in skeletal muscles and an increase in energy expenditure. It indicates that osteocalcin can increase muscle work by increasing insulin sensitivity. Elucidation of how OC/Gprc6a signaling promotes β-cell proliferation may provide a novel approach for diabetes treatment (Wei et al., 2014).

### GPR119 AND S1P RECEPTOR

G protein-coupled receptor 119 (GPR119) is expressed in the pancreas in rodents and humans. A selective small molecular GPR-119 agonist, PSN632408, can reduce food intake and body weight gain in rodents through increasing intracellular cAMP levels (Overton et al., 2006). PSN632408 and an endogenous ligand of GPR119, oleoylethanolamide (OEA), can stimulate β-cell replication in mouse islets *in vitro*. OEA and PSN632408 improved mouse islet graft function in diabetic mouse insulin-positive/BrdU-positive β-cells. OEA and PSN632408 treatment increased active GLP-1 levels in mice plasma (Gao et al., 2011). The ability of PSN632408 to stimulate β-cell replication in cultured mouse islets and *in vivo* has been recently demonstrated (Ansarullah et al. 2013).

Many of the immune suppressive drugs are toxic to β-cells. Because of this, their clinical administration after islet transplantation for type 1 diabetes was limited. However, Truong and collegues found that FTY720, an immune suppressor that modulates sphingosine-1-phosphate receptor (S1PR, a G protein-coupled receptor) activity, did not impair human islet function *in vitro* or *in vivo* (Truong et al., 2007). Interestingly, treatment with FTY720 can prevent the onset of diabetes in an animal model of human type 1 diabetes by activating immune cells in the lymph nodes (Jörns et al., 2010). Moreover, oral administration of FTY720 to obese mice can increase β-cell mass and blood insulin levels. This function is mediated by decreasing the cyclin-dependent kinase inhibitor p57 (KIP2) level, and at the same time, increasing the cyclin D3 level (Zhao et al., 2012). By inhibiting β-cell apoptosis, FTY720 can retain β-cell mass and prevent damage of pancreatic islet (Moon et al., 2013). Through finding ultra-structural changes in pancreatic β-cells after treatment with anti-TCR and FTY720 in type 1 diabetic rats, a similar improvement of β-cell viability has been observed (Jörns et al., 2014).

### GLUCOKINASE ACTIVATORS

Glucokinase (GK) activator is effective in lowering blood glucose concentration not only by the enhancement of glucose uptake in the liver but also by the secretion of insulin from pancreatic β-cell (Park, 2012). Activation of glucokinase by small chemical compound promotes pancreatic β-cell proliferation. When treated with GKA50, a GK agonist, INS-1 β-cell proliferation increased at basal levels of glucose. This effect is mediated by the IRS-2/PI3K/PKB pathway. Moreover, GKA50 was found to prevent INS-1 cell apoptosis under the impairment of chronic high glucose conditions (Wei et al., 2009). YH-GKA, another GK activator, also increased the INS-1 β-cell number by up-regulating IRS-2 and subsequently activating AKT/PKB. IRS-2 down-regulation can decrease the proliferation effect of YH-GKA. YH-GKA induces ATP content and citrate synthase activity which blocks β-cell apoptosis (Oh et al., 2014). Importantly, GKA was shown to be sufficient and effective in promoting β-cell proliferation in mice (Salpeter et al., 2010). Positive impact of GK agonists on promoting β-cell proliferation and preserving β-cell mass has been shown in aging mice and diabetic rat models (Stolovich-Rain et al., 2012; Futamura et al., 2012).

### OTHER FACTORS

Betatrophin has been shown to induce β-cell proliferation in a mouse model of insulin resistance on the basis of gain-of-function evidence derived from over-expression of betatrophin in the mouse liver (Yi et al., 2013). Betatrophin is mainly expressed in the liver and fat and its plasma level is associated with β-cell proliferation in insulin resistance mice and the mouse model during gestation. Treatment with an insulin receptor antagonist S961 elicited insulin resistance and led to an incremental quantity of betatrophin. Blocking the insulin receptor with a high dose of S961 led to the mice glucose intolerant and an increase in β-cell replication. However, a recent study using both betatrophin knockout and over-expression approaches indicate that betatrophin does not control β-cell expansion (Gusarova et al., 2014). β-Cells from individual mice appear to have a broad range of responses to betatrophin and redundancy may exist that could compensate for the loss of betatrophin function (Yi et al., 2014). In any event, further investigation is needed to clarify whether betatrophin can indeed play a role in regulating β-cell expansion together with other factors.

Some other factors have been reported to be able to induce β-cell proliferation. Early studies revealed that a lectin from *Agaricus bisporus* (mushroom) (ABL) causes a dose-dependent inhibition of tumor cell proliferation (Yu et al., 1993; 1999). Surprisingly, a recent research found that ABL administration promoted β-cell proliferation (Wang et al., 2012). It is unclear how ABL has opposite effects on tumor cell and β-cell proliferation.

The IGF-1 receptor (IGF1R) has become a therapeutic target for cancer treatment. The efficacy of OSI-906, a dual inhibitor of IGF1R and insulin receptor, was found to elicit β-cell proliferation to increase β-cell mass in male mice (Shirakawa et al., 2014). While insulin signaling in β-cells was not affected by OSI-906, how OSI-906 treatment leads to β-cell expansion needs to be further investigated.

Using a high throughput primary β-cell replication assay, two adenosine kinase (ADK) inhibitors, 5-Iodotubercidin and ABT-702, have been identified and shown to increase β-cell mass (Annes et al., 2012).

Finally, a unique compound epoxypukalide was reported to induce a 2.5-fold increase in β-cell proliferation, through activation of the ERK1/2 signaling pathway and up-regulation of Cyclin D2/Cyclin E. Epoxypukalide did not attenuate glucose-stimulated insulin secretion in rat islets (López-Acosta et al., 2013). The mechanism of epoxypukalide action remains to be elucidated.

## CONCLUDING REMARKS

Until now, there is no promising medication for expanding β-cell mass for diabetic treatment. However, strong evidence has been accumulated to support that β-cell proliferation could be enhanced by small chemical compounds or extracellular factors in animal models (Fig. 1). As β-cell proliferation was detected in a surgically resected pancreas from an 89-year-old with recent-onset diabetes (Meier et al., 2006), this observation encourages further exploration of strategies to promote adult β-cell expansion as a therapeutic approach for treatment of diabetes.

Clearly much more efforts are needed to identify small molecules and protein factors that can explicitly elicit human pancreatic β-cell regeneration. Along this line, it is important to keep in mind that significant differences exist between human and other animals such as mice and rat. Therefore, tests must be further conducted with β-cells in cultured human pancreatic cell lines or islet tissues if a factor is initially discovered and studied in animals. Moreover, any potential therapeutic factors should be able to effectively target β-cells *in vivo* to minimize possible side effects.

Because cell proliferation is typically regulated through intercellular signaling, many growth factors and hormones have been tested for their ability to influence β-cell proliferation. For example, osteocalcin produced by osteoblasts in the bone acts as a hormone to stimulate β-cell proliferation in the pancreas (Wei et al., 2014). Similarly, there are other hormones such as prolactin and thyroid hormone increased in gestation, and leptin increased in adiposity to elicit β-cell proliferation.

While it is challenging to identify novel substances that increase β-cell mass, efforts have been made to test some existing medications such as antioxidants, immune-suppressants, and even anti-cancer drugs, for their effect on β-cell expansion. Interestingly, an inhibitor for both IGF receptor and insulin receptor can effectively expand β-cell mass (Shirakawa et al., 2014). It remains to be clarified as to how this inhibitor of IGF/insulin signaling, a potent growth-promoting pathway, acts to enhance β-cell proliferation and survival. Not surprisingly, most of these drugs exhibited shortcomings with regards to their specificity and efficiency.

Currently, most of the β-cell research focuses on individual factors for their effect on β-cell expansion. However, β-cell expansion occurs in a complex patho-physiological background and therefore, multiple factors should be considered to simultaneously interfere with several steps or pathways to enhance β-cell proliferation. For example, a recently discovered novel hormone, irisin, was deemed to cross-talk with betatrophin in the process of β-cell regeneration and dedifferentiation (Boström et al., 2012; Sanchis-Gomar and Perez-Quilis, 2014; Zhang et al., 2014). It would be interesting to learn how such inter-molecular and inter-pathway interactions affect β-cell expansion.

Notably, β-cells can proliferate while retaining their differentiated phenotypes *in vivo*. This fact provides another facet to reflect the complexity of β-cell proliferation *in vivo*. Given all that, in the future we might need a “cocktail” program to realize the efficiency and safety of β-cell regeneration for dealing with diabetes. Although we are facing a hard situation, obviously, we are on the right path.

## ABBREVIATIONS

ADK: adenosine kinase
GABA: γ-aminobutyric acid
GK: Glucokinase
GLT-1: glutamate transporter-1
OC: osteocalcin
OEA: oleoylethanolamide
TFF2/3: trefoil factors 2/3
